# Supplementary material for: SLC25A12 Missense Variant in Nova Scotia Duck Tolling Retrievers Affected by Cerebellar Degeneration—Myositis Complex (CDMC)
Source: Genes (Basel). 2022 Jul 9;13(7):1223. doi: 10.3390/genes13071223 (PMC9319113; doi:10.3390/genes13071223)
Supplement: Supplementary file 1 [file genes-13-01223-s001.zip › S1_Figure.pdf]

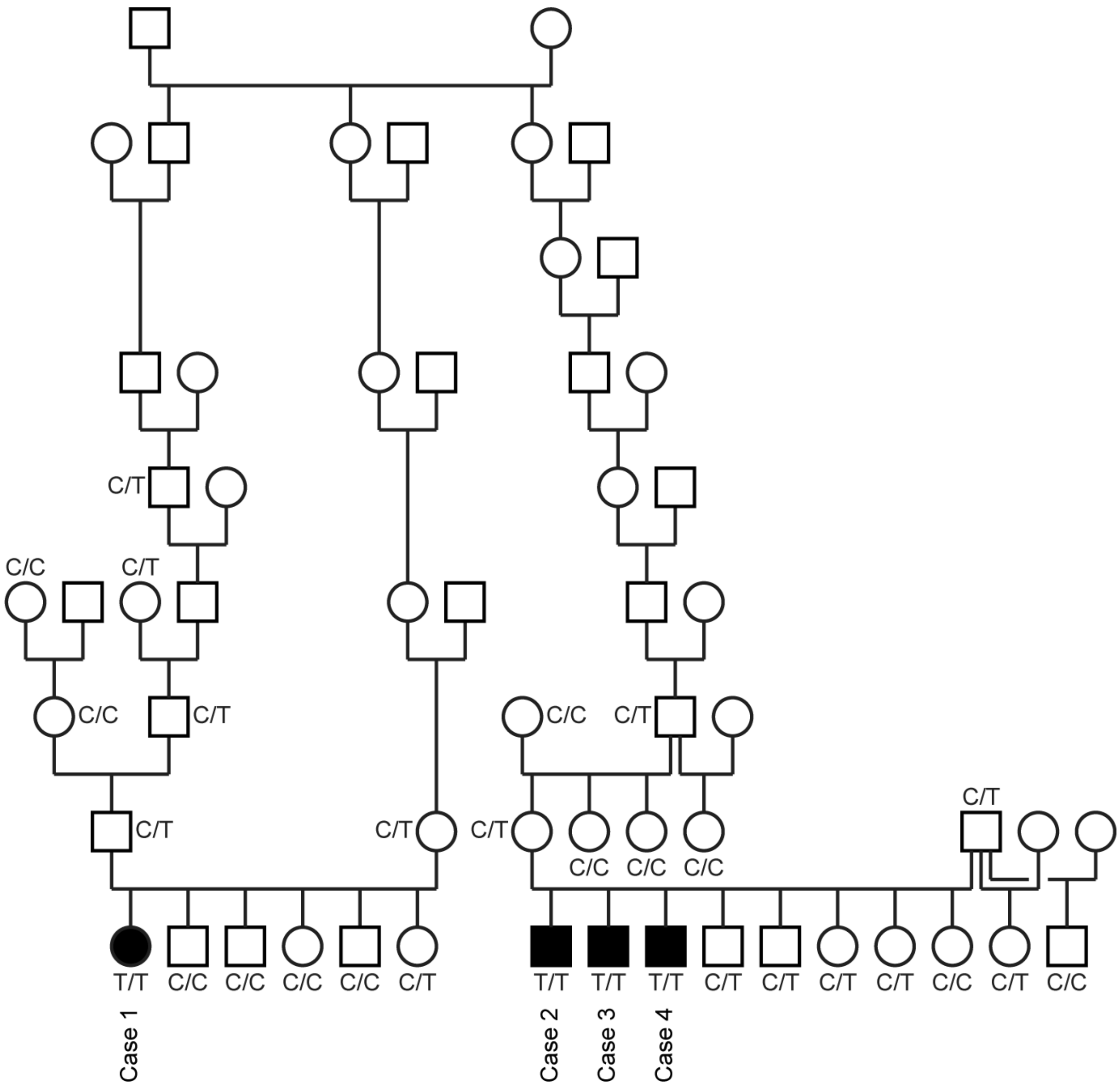

S1 Figure. Pedigree of NSDTR dogs with cerebellar degeneration and myositis complex (CDMC). Affected dogs are indicated with filled symbols. The index family with one affected dog is shown at the bottom left of the pedigree. Genotypes at the *SLC25A12*:c.1337C>T variant are indicated for all dogs, from which a DNA sample was available. During the investigation, an additional second litter with three affected puppies became available (bottom right). Common ancestors could be identified for three of four parents of the affected litters. The pedigree is compatible with a monogenic autosomal recessive inheritance of the trait.
